# Does the day of the week predict the presence of microbiologically confirmed ventilator-associated pneumonia?

**DOI:** 10.1186/cc10670

**Published:** 2012-03-20

**Authors:** C Linssen, H Van Dessel, W Van Mook

**Affiliations:** 1 Maastricht University Medical Centre, Maastricht, the Netherlands

## Introduction

At our hospital, ventilator-associated pneumonia (VAP) is diagnosed by microbiological and cytological analysis of bronchoalveolar lavage fluid (BALF). Opening hours of the in-house microbiological laboratory are between 8:00 am and 5:00 pm. During off-hours a laboratory technician is on call for urgent samples including BALF. The total laboratory work-up of the BALF takes 2 hours. The aim of the present study was to detect patterns in the submission time of BALF samples.

## Methods

During a 60-month period (January 2006 to December 2010), the day and hour of submission of all consecutive BALF samples obtained from patients suspected of VAP were recorded. VAP was microbiologically confirmed if quantitative cultures were ≥10^4 ^cfu/ml and/or presence of ≥2% infected cells.

## Results

A total of 376 BALF samples were included. On weekdays, on average a total of 59.8 ± 11.4 were submitted, compared to 34 and 43 samples on Saturdays and Sundays. For more than one-half (203, 54%) of the samples, the on-duty laboratory technician was required: 86 (23%) samples arrived within 1 hour before closing time, and an additional 117 (31%) were submitted thereafter. VAP was diagnosed in 149 (39.6%) samples, of which 79 (53%) after closing hours. BALF samples were obtained more frequently on Thursdays and Fridays (51 and 47 samples respectively) compared to Mondays and Tuesdays (64 and 76 samples). Interestingly, VAP was confirmed proportionally more frequently on Mondays and Tuesdays (26/51 (51%) and 23/47 (49%)) compared to Thursdays and Fridays (20/64 (31%) and 26/76 (34%)).

## Conclusion

The high number of BALFs processed after laboratory opening hours is of concern because of the suboptimal working conditions (fatigue, lack of supervision and experience). Technicians' time spent on these samples puts a strain on the laboratory in terms of costs and absence of the technicians because of legal recuperation. A higher number of confirmed episodes of VAP early in the week compared to just before the beginning of the weekend, combined with a larger number of BALF samples obtained on Thursdays and Fridays, may suggest that clinicians want to exclude VAP before the weekend resulting in a lower threshold for requesting a BALF.

